# Fusion and classification algorithm of octacalcium phosphate production based on XRD and FTIR data

**DOI:** 10.1038/s41598-024-51795-0

**Published:** 2024-01-17

**Authors:** Mauro Nascimben, Ilijana Kovrlija, Janis Locs, Dagnija Loca, Lia Rimondini

**Affiliations:** 1 Center for Translational Research on Autoimmune and Allergic Diseases-CAAD, Department of Health Sciences, Università del Piemonte Orientale UPO, 28100 Novara, Italy; 2 Enginsoft SpA, 35129 Padua, Italy; 3 Institute of Biomaterials and Bioengineering, Faculty of Natural Sciences and Technology, Riga Technical University, Riga, Pulka 3, LV-1007 Latvia; 4 Baltic Biomaterials Centre of Excellence, Headquarters at Riga Technical University, Riga, Latvia

**Keywords:** Biomedical materials, Biomaterials, Biosynthesis, Machine learning

## Abstract

The present manuscript tested an automated analysis sequence to provide a decision support system to track the OCP synthesis from $\alpha$-TCP over time. Initially, the XRD and FTIR signals from a hundredfold scaled-up hydrolysis of OCP from $\alpha$-TCP were fused and modeled by the curve fitting based on the significantly established maxima from the literature and nine features extracted from the fitted shapes. Afterward, the analysis sequence enclosed the machine learning techniques for feature ranking, spatial filtering, and dimensionality reduction to support the automatic recognition of the synthesis stages. The proposed analysis pipeline for OCP identification might be the foundation for a decision support system explicitly targeting OCP synthesis. Future projects will exploit the suggested methodology for pinpointing the OCP production over time (including the intermediary phases present in the OCP formation) and for evaluating whether biological variables might be merged with biomaterial properties to build a unified model of tissue response to the implant.

## Introduction

Bone regeneration is crucial in multiple healthcare sectors, such as dentistry and orthopedics, where bone replacement and healing are fundamental for a patient’s well-being and quality of life. Regenerative medicine’s primary strive is osteogenesis, the bone formation and growth process. Osteogenesis is stimulated by osteoinduction, which involves recruiting and stimulating stem cells to promote their differentiation into preosteoblasts^1^. On the contrary, osteoconduction is the ability of bone-forming cells to advance across a matrix and partly replace it with the new bone over a certain period^2^. However, when it comes to implants, osteoconduction is also dependent on the conditions within the setup and the reactions to the used biomaterial. As the final goal is to have a material that shows osteoinductive properties (e.g., calcium phosphates, CaPs), it is essential to steer the research toward finding the best candidates^3^. Besides having the properties mentioned above (biocompatibility, osteoconductivity, osteoinductivity), CaPs have structural and compositional features similar to native bone and are abundantly present in the human body. The spectra of present CaP compositions are very diverse^4^, with plentiful potential phases (alongside apatite) able to form depending on experimental conditions, experimental mistakes (approximations), reactions with the immersion solutions, etc.^5^.

One of the CaPs that stood out, based on its structural similarity to hydroxyapatite and extraordinary biological attributes, is octacalcium phosphate (OCP)^6^. An additional advantage of OCP is its ability to convert to the thermodynamically more stable phase (hydroxyapatite), both in vitro and in vivo^7^. It is generally synthesized through the precipitation of various aqueous solutions containing calcium and phosphate ion source or via the hydrolysis of $\alpha$-tricalcium phosphate ($\alpha$-TCP)^8,9^ or brushite (dicalcium phosphate dihydrate, DCPD)^10,11^. Moreover, OCP can stimulate bone formation by osteoblast differentiation and osteoclast formation. However, the stability of the pure OCP phase formation depends on a narrow region of pH and temperature of the reaction. At the same time, those parameters can influence the crystallinity, size, and morphology of the crystals, as well as possible conversion to a different CaP phase upon the end of the synthesis^12–14^. Consequently, synthesizing and characterizing the properties of engineered biomaterials require extensive laboratory experiments not only for manufacturing but also to study the biocompatibility between the physicochemical properties of the surface with the surrounding biological microenvironment^15^. Development procedures should establish and encompass protocols that could assess at what stage the synthesis is or what could be the potential end product, without the experimental guidelines relying primarily on empirical methods or researchers’ intuition (e.g., trial-and-error). One potential solution could be that the exploitable data from the pre-clinical phase is used for building computational models able to support decision-making during biomaterials design^16^. In the early stages of materials synthesis for tissue engineering, data from in vitro assays analyzed with advanced data mining methods could provide innovative information for optimizing the composition of the biomaterials^17–19^. For example, the bacterial proliferation on polymeric material was determined through machine learning (ML)^20^, or Wang et al. studied fatigue cracks on metallic implants^21^ through automated algorithms. Another advantage could be the application of predictive techniques on multi-dimensional data to automate or bolster peculiar phases of the manufacturing process. ML processes are automated compared to the classic design of experiments (DoE) optimization in bioengineering, where an operator has to decide on a limited set of input parameters for the model. The ML algorithms disclose relevant patterns in the data providing high-accuracy prediction or categorization^22^. They are preferred over DoE when datasets are not small or not composed of pure numerical values, given the ability of ML to handle different data formats such as images, spectra, numerical datasets, categorical variables, etc. Indeed, biomedical implant production evaluated through data analytics could overcome the limitations of physics modeling, commonly applied to simulate an output given input and standard pre-selected environmental parameters, offering effective surrogate methods for materials chemistry^23^.

In the present research, data collected from the synthesis of octacalcium phosphate from $\alpha$-tricalcium phosphate ($\alpha$-TCP) based on the X-ray diffractometry (XRD) and Fourier transform infrared spectroscopy (FTIR) has been merged and analyzed by using a novel computational model. The paucity of data on how to obtain a pure OCP phase while at the same time maintaining the successful scale-ups of the synthesis or time alterations shows a dire need for help that artificial intelligence can provide. This multi-source approach that integrates data from different laboratory techniques and computational modeling might help characterize OCP formation more extensively. Other authors already tried to merge data, for example, to enhance comprehension of crystallized membrane proteins; however, their approach fused 2D crystal images from the same source^24^. The current investigation aims at proposing a data mining methodology or analysis sequence whose main aspects are outlined as follows:

- explore the possibility of merging the information extracted from heterogeneous data sources recorded during the scaled-up OCP synthesis.
- evaluate the most relevant features derived from the peak modeling of the recorded XRD and FTIR signals through machine learning.
- facilitate the interpretation of the outcomes during OCP manufacturing by proposing an algorithm able to define OCP formation phases automatically. Actual implementation represents the foundation for a more complex system based on the same logic that can potentially track all intermediary phases that may occur during the OCP formation.

The final goal is to support researchers’ decision-making by offering supplementary analysis to existing laboratory practice.

## Results

The analysis sequence considered the features derived from modeling the peaks belonging to the scaled-up XRD and FTIR signals. The scaled-up synthesis (10 g final product amount) was selected as the small-scale one (e.g., 100 mg, which was the target of previous research^9^) resulted in quite a small yield of the final product; thus, the reproducibility and uniformity between different batches was not ensured. Moreover, to accomplish a multi-technique characterization across various platforms and to later use considerable amounts of the product for in vitro and in vivo tests, high yield and scale-up of the technology are of utmost importance.

**Figure 1:**
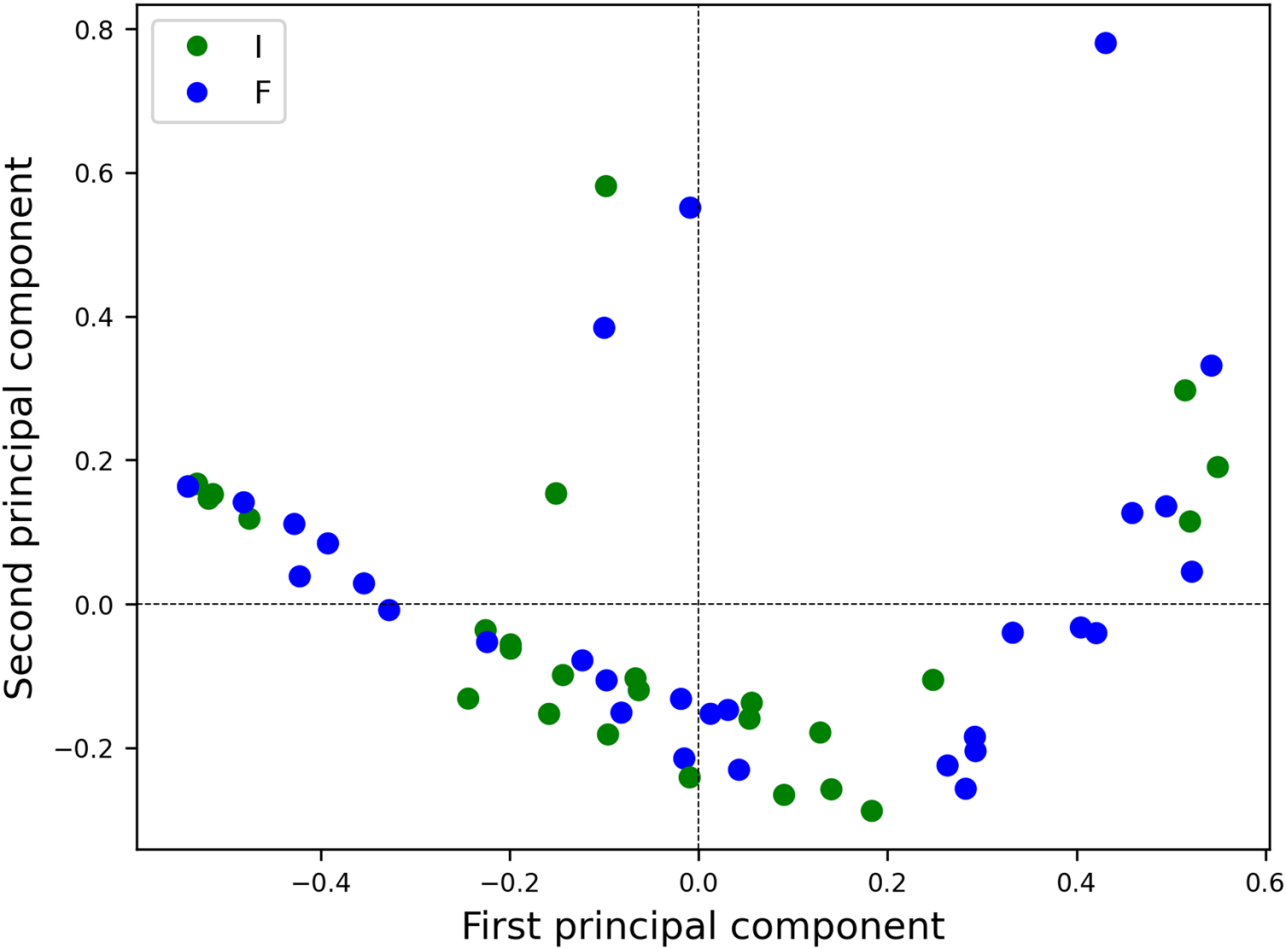
Low dimensional representation through kernel principal component analysis of the seven most important features (“I” are green points depicting “Initial” phase, whereas “F” are blue features of “Final” stage).

After the XRD and FTIR shapes were processed to compatible signals, nine features were derived from the modeled peaks through Gaussian or Lorentzian shapes. Recursive feature elimination determined a subset of relevant attributes from the nine collected descriptors. This subset of seven attributes of each signal peak was the dataset employed to recognize OCP production phases. A low-dimensional embedding of each peak’s seven most significant descriptors was computed for visualization purposes through Kernel Principal component analysis (k-PCA) employing a cosine template (Fig. 1): the two classes representing the time evolution of OCP production overlap, making it challenging to identify the different production stages.

**Figure 2:**
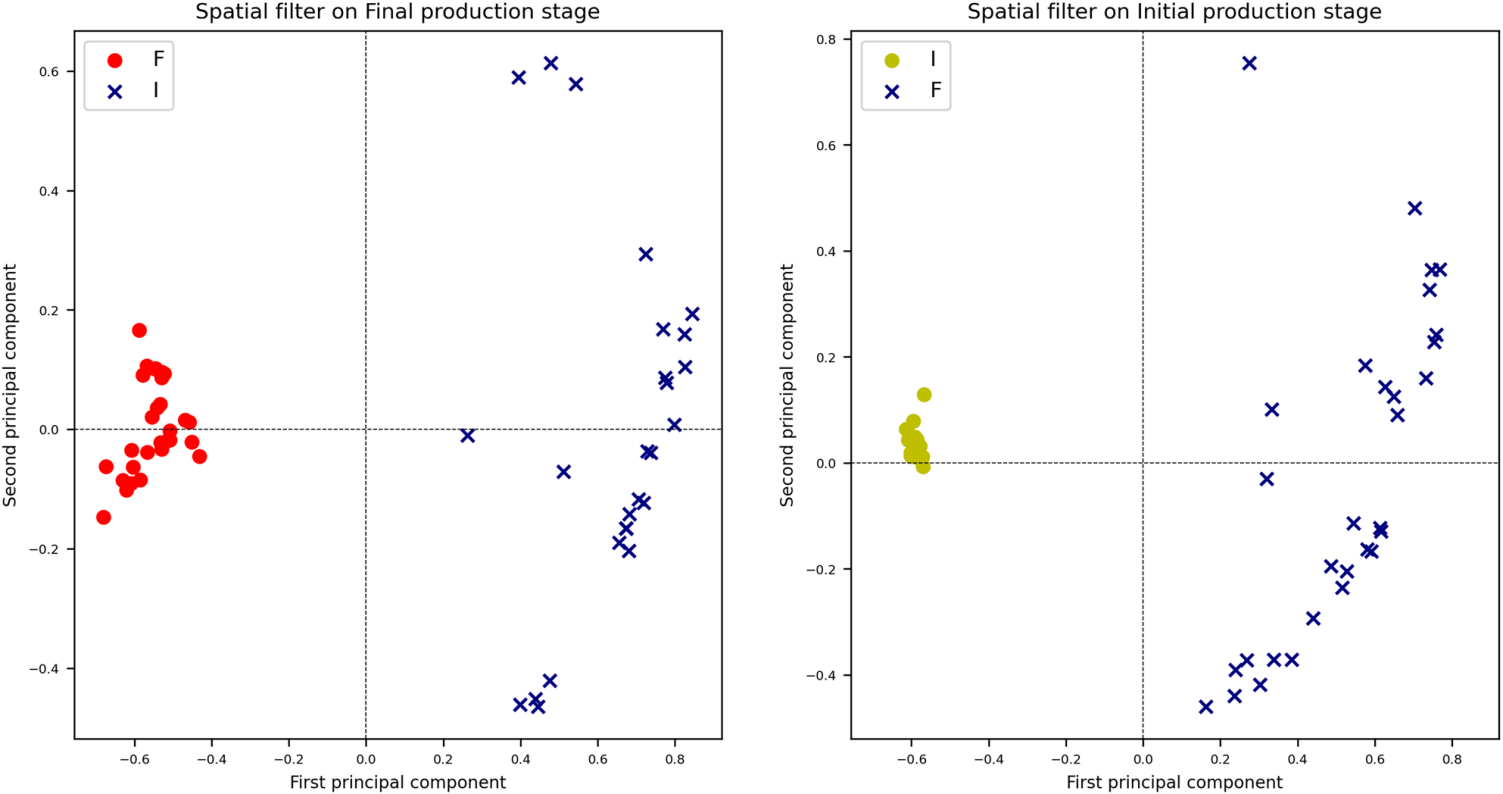
Kernel principal component analysis of the spatially filtered classes in the one-vs-other configurations. On the left, the “Final” spatially filtered and k-PCA embedded class is shown in red versus the class I. On the right, the “Initial” spatially filtered and k-PCA reduced samples are in yellow.

Undeniably, an algorithm working as an automatic scorer might need further data processing to learn how to associate an XRD or FTIR value to one of the two classes representing OCP (analogous to “F” class) or $\alpha$-TCP (corresponding to “I” class). A solution could be transforming the values to facilitate the automatic identification of OCP formation’s initial and final stages. For example, each class could be rotated concerning the other to occupy different portions of the Cartesian plane; in this way, the classes could create a non-overlapping map with well-defined point clouds. One approach for reducing data structure dependence could be applying spatial filters^25–28^ paired with a transformation highlighting the presence of clusters. These two methods applied in sequence to the data might facilitate the identification of a decision boundary for scoring OCP production phases. Spatial filters are commonly used for noise reduction on images^29,30^ or as part of the first layers of convolutional neural networks or other machine learning analysis sequences^31,32^. Other applications include improving Gaussian peak shape determination in optics^33^. The spatial filter enhanced class separability by uncorrelating the one-versus-other class configurations. In Fig. 2, kernel principal component analysis reduced the seven spatially filtered features to highlight the patterns useful for automatic OCP phase prediction. Indeed k-PCA can mimic the behavior of spatial clustering algorithms^34^, ameliorating the separability between classes’ instances.

**Figure 3:**
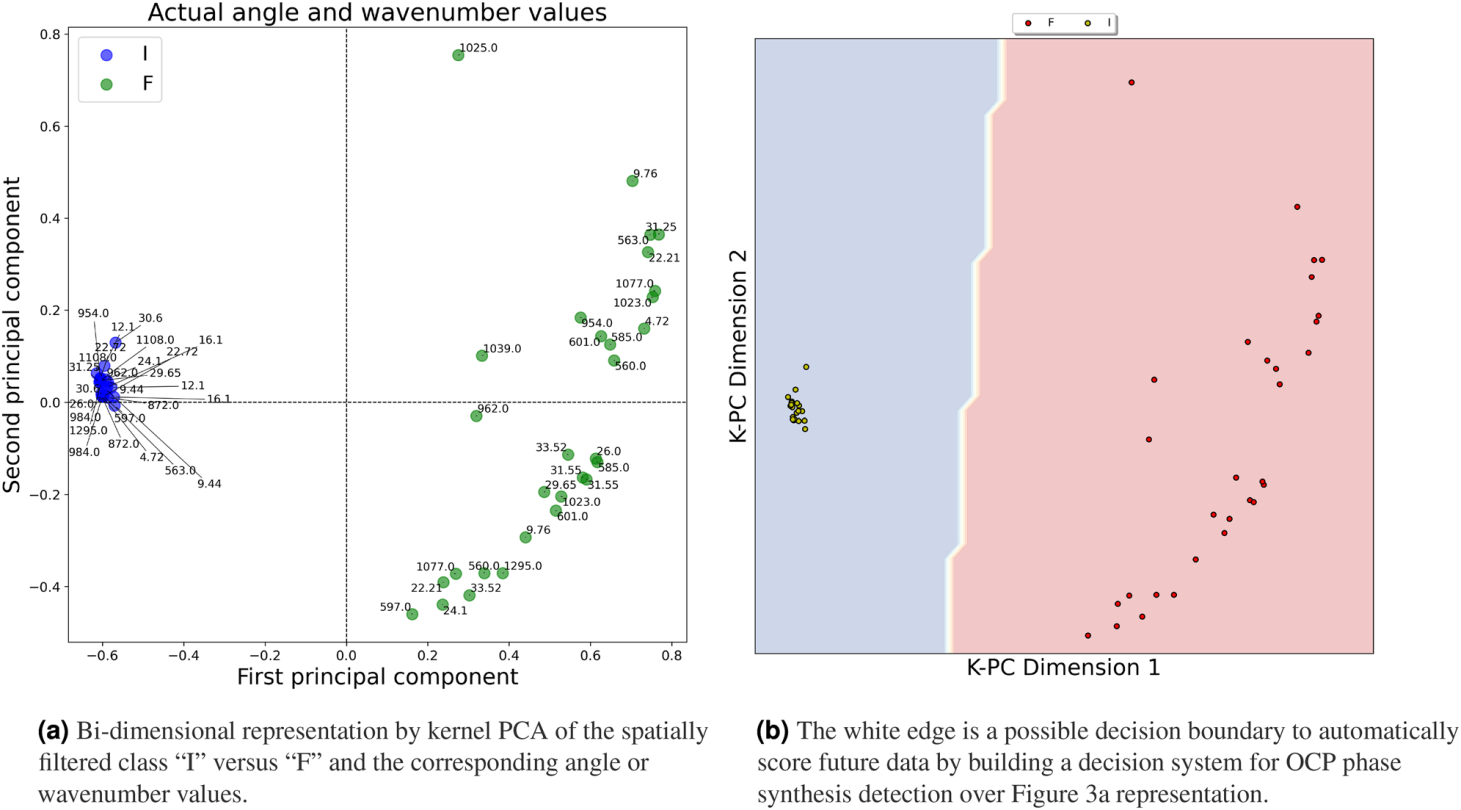
Application of the spatial filter followed by dimensionality reduction using k-PCA highlighting model usage and its interpretability.

The final map of one spatially filtered class (e.g., I) versus the other is shown in Fig. 3a; the two clusters produced by k-PCA were also associated with each theta or wavenumber to link XRD and FTIR signal characteristics. By applying this computational strategy, the production phases could be represented as two-dimensional embeddings, easily interpretable by a human operator for quality control. Furthermore, presenting two distinct point clouds is crucial to draw a decision boundary exploitable for building a support decision system to categorize the OCP production phases. After applying the spatial filter and reducing the dimensionality by k-PCA, a linear boundary could be sufficient to classify the dataset instances automatically, as demonstrated graphically in Fig. 3b. According to the example, points falling on the right side of the edge could be classified as “F”, whereas those laying on the left of the white boundary are marked as the “I” OCP phase. Table 1 reports the modifications of the variance found in the First and Second k-PCA components when the data is spatially filtered (e.g., Fig. 2) or not (e.g., Fig. 1).

**Table 1:** Variance modifications in the low dimensional embedding by k-PCA with or without spatial filtering.

	Variance of k-PCA components	
	First component	Second component
Class I	0.0902	0.0421
Class F	0.1138	0.0573
Class I (spatially filtered)	0.0002	0.0008
Class F (spatially filtered)	0.0036	0.006

Understanding and accounting for the common spatial patterns in the fused data makes it possible to reduce the overall variance, leading to more accurate and reliable analyses and predictions. The peak shape descriptors composing the dataset analyzed through the proposed procedure were transformed to enhance specific features of the signals, making it easier to detect data patterns or structures. In conclusion, the research objective of obtaining two separable point clouds representing the distinct production phases of OCP synthesis was reached by applying spatial filtering paired with k-PCA dimensionality reduction to XRD and FTIR peaks descriptors. An automated decision support system might exploit the current analysis sequence to track OCP production phases consistently.

## Discussion

Machine learning-based procedures can support materials science, offering techniques to accelerate innovation and the return on investment (laboratory and experimental costs). In the current investigation, we proposed merging heterogeneous data sources from XRD patterns and FTIR spectra to augment the information available for accurate and automatic identification of octacalcium phosphate production phases. The innovation resides in the possibility of tracking the different stages of biomaterial synthesis by fusing standard laboratory tests. For the development of biomaterials, multiple steps to determine the physicochemical characteristics accurately and comprehensively are needed. Thus, combining various sources in a unique algorithm might propose a different approach than analyzing the physicochemical properties of each laboratory test separately. The complicated interaction between structures, composition, and hands-on experience is a significant step to overcome in clinical biomaterials design. Fusing sources of information to define these aspects might improve the understanding of biomaterials production.

In addition to proposing a way to merge signals from different sources (XRD and FTIR), this study introduced a novel analysis sequence to extract and evaluate features characterizing the initial and final stages of octacalcium phosphate synthesis. The analysis pipeline included a spatial filter followed by dimensionality reduction with k-PCA, demonstrating how this combination of techniques could produce unique patterns describing biomaterial production. It could constitute the foundation for a more sophisticated methodology that could be developed to track the kinetics of OCP phase formation, including the intermediate states. As proven in^35^, the middle stages of $\alpha$-TCP to OCP conversion contain the brushite phase (dicalcium phosphate dihydrate, DCPD), a precursor to obtaining pure octacalcium phosphate. Unfortunately, even though DCPD is clearly seen in XRD patterns, FTIR showed trace instability and is more subtle to recognize without supervision^36,37^. Even though the XRD patterns are the primary way to identify the crystalline phase (in conjunction with the International Centre for Diffraction Data PDF-2 (ICDD) database), due to the specific crystal structure of OCP that is very similar to hydroxyapatite, certain parts of the pattern overlap and it is not possible (yet) to differentiate OCP from HAp with certainty from using only XRD patterns. In order to finalize the structural identification, FTIR spectroscopy was conducted on all samples. From the point of view of materials science, the advantage of the proposed methodology for OCP production tracking (as it is a metastable phase with high similarity to HAp) is the possibility of showing the chemical composition into a well-defined spatially 2D embedding of the original features representing the detection of a specific vibration of hydrogen phosphate ($\hbox {HPO}_{4}^{2-}$) ion. Pinpointing the presence of $\hbox {HPO}_{4}^{2-}$ and phosphate ($\hbox {PO}_{4}^{3-}$) groups is paramount in determining OCP phase purity. OCP’s characteristic vibrations at 917, 875, 1007, and 1295 cm$^{-1}$ show the presence of hydrogen phosphate, and they differentiate the OCP from stoichiometric HAp. Thus, combining XRD and FTIR is crucial for OCP production tracking, as demonstrated in the proposed analysis pipeline. Indeed, only peaks for OCP and $\alpha$-TCP were included during the current investigation. In the future, the algorithm developed within this study will be fine-tuned to follow all relevant kinetic steps of CaP transformation.

Additionally, fusing different data sources can significantly enhance the predictive capabilities of machine learning models for several reasons. One is that by integrating multiple data sources, researchers can access a more comprehensive view of the problem or phenomenon they are trying to model. This broader perspective can provide more context, enabling a more accurate understanding of the underlying patterns and relationships within the data. Different data sources may offer diverse sets of features that can complement each other. Combining these features allows researchers to create a more robust and informative feature set for training the machine-learning model; this leads to improved feature representation and a better understanding of the underlying data structure. Another advantage is connected with a reduced bias and variance. Incorporating data from various sources can help mitigate the biases and variances in individual data sets. By leveraging diverse data, a researcher can balance out any tendencies that might be present in one data source, leading to a more balanced and accurate model. Moreover, an improved generalization ability could arise from merged datasets. Fusing data from multiple sources can enhance the generalization capabilities of a machine-learning model. By training on diverse data, the model can learn more generalized patterns and relationships, making it more robust and capable of making accurate predictions on unseen data.

Another relevant aspect of the current work is the application of spatial filtering techniques on peak descriptors to ease the automatic scoring of the initial and final phases of OCP synthesis from $\alpha$-TCP. This addition, together with k-PCA dimensionality reduction, improves the algorithm’s ability to detect the production stages without supervision by creating well-defined clusters, consequently improving the accuracy of a classifier trained to label the merged data. Through spatial filtering, similarities or shared characteristics existing within neighboring data points are recognized: for example, trends, clusters, autocorrelation, and other forms of interdependence. Identifying common patterns can reduce variance in the data, leading to more robust and insightful predictive analyses. Modifying the spatial filter formulation to work in a one-versus-rest configuration could make scoring more than two OCP production phases possible.

Even if the procedure looks promising, a limitation of the current study is connected with the ability to test it on other bioengineered materials. Although the analysis pipeline at the end could identify suitable clusters tracking biomaterial compositional modifications, as shown in Fig. 3b, the procedure might be evaluated in synthesizing other compounds to test its effectiveness.

A final remark and a possible research direction for furtherly expanding current results could be the inclusion of biological variables. The microenvironment enclosing cells and biomaterials implanted in the human body requires the control of cell function by manipulating surface properties to shape the biological responses (e.g., cell phenotypes). The proposed technique could incorporate material properties with biological information to tune cell behavior in response to modifications in the materials’ biophysical properties. For example, the biomaterial’s mechanical properties influence the development of fibrosis; thus, linking the material’s properties to cell activity might be another advancement in biomaterials research. Future research will explore the possibility of merging biological and material compositional variables.

## Methods

**Figure 4:**
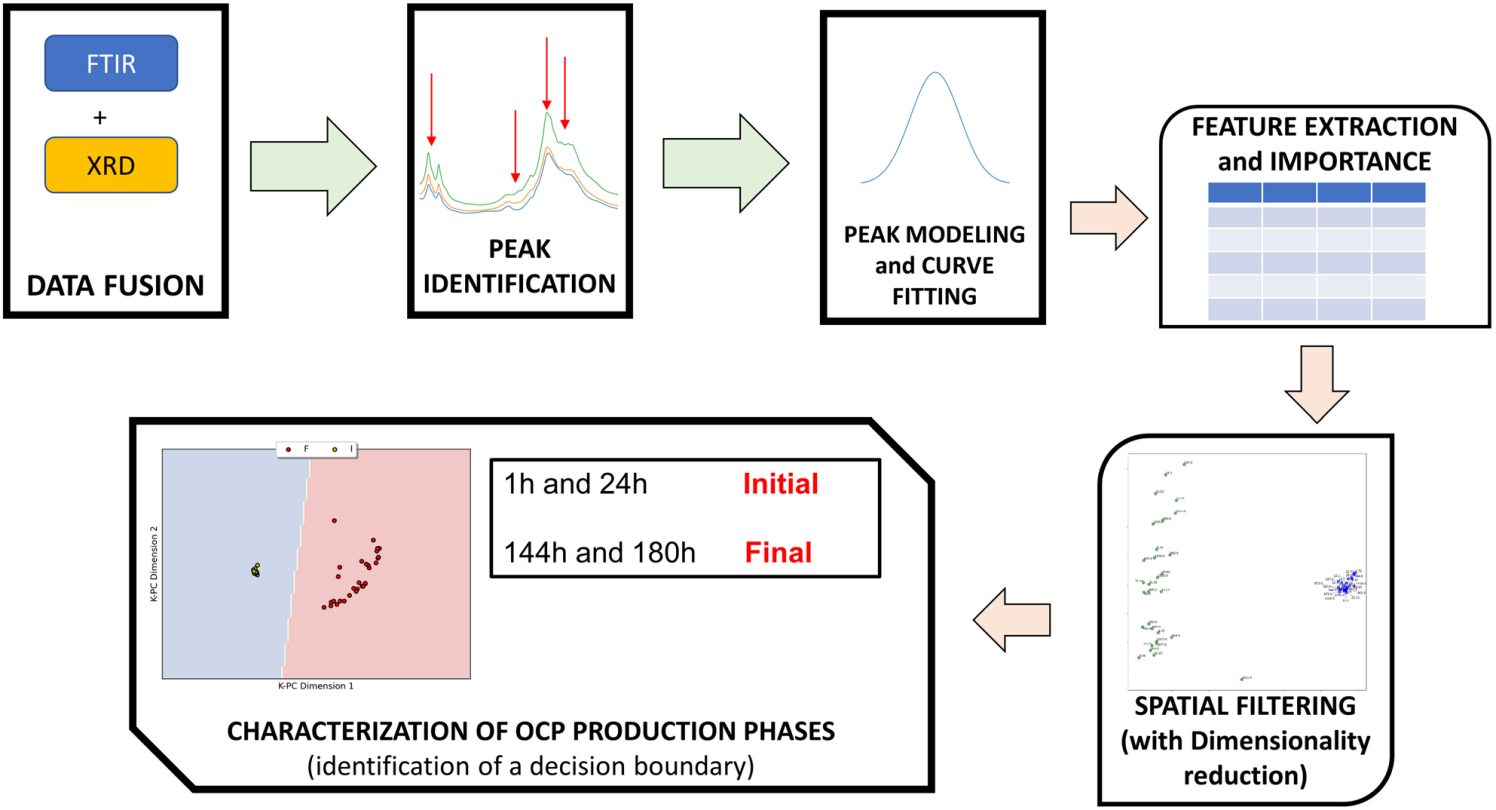
Green arrows: initial sequence of the proposed analysis pipeline with pre-processing of the FTIR and XRD signals and their fusion, automatic peak detection, modeling of the peaks. Orange arrows: feature extraction and feature importance evaluation by machine learning, application of a spatial filter paired with kernel principal component analysis on the the reduced dataset, theoretical decisin boundary to distinguish between the stages of OCP synthesis.

The analysis pipeline tested in the current investigation is summarized in Fig. 4. The initial analysis steps focused on merging XRD patterns and FTIR spectra to enlarge the dataset’s size and test if these joint attributes might reveal critical aspects of OCP synthesis. Afterward, the signals’ peaks were modeled as Gaussian or Lorentizian shapes, and a set of features or ratios was calculated, acting as $\alpha$-TCP or OCP descriptors.

Once the most important and descriptive features were obtained by recursive feature elimination, they were related to OCP synthesis stages (“Initial” or “Final”) to verify if the current methodology could automatically highlight characteristics of OCP production. The proposed solution exploited a spatial filter paired with dimensionality reduction, demonstrating that it can automate the OCP or $\alpha$-TCP discrimination.

### Octacalcium phosphate synthesis and characterization: laboratory data

OCP was obtained from low temperature $\alpha$-tricalcium phosphate ($\alpha$-TCP) via hydrolysis method. The scaled-up synthesis (100 times) was used to evaluate the quality of the product. Briefly, 10 g of $\alpha$-TCP were placed in 5 L of 0.0016 M orthophosphoric acid ($\hbox {H}_{3}\hbox {PO}_{4}$, 75$\%$ Latvijas Kımija, Riga, Latvia) and stirred with an overhead mixer (500 rpm) during the course of 180 h. The pH was monitored throughout the entire duration of the synthesis. The collected suspensions were centrifuged at 3000 rpm for 2 min, washed with deionized water, and dried at 37 $^{\circ }$C. During the experiment, multiple samples from the reactor were collected to follow the compositional changes after 1 h, 24 h, 30 h, 48 h, 78 h, 96 h, 144 h, and 180 h. In the present analysis, the first two (i.e., 1 h and 24 h) and the last two (144 h and 180 h) XRD and FTIR signals were included in the numerical experiments.

The presence of crystalline phases was examined by using X-ray powder diffractometry (XRD) and Fourier-transform infrared spectroscopy (FTIR). XRD was performed using PANalytical Aeris diffractometer (The Netherlands) and accompanying analyses were performed with suitable software (X’Pert Data Collector, X’Pert Data Viewer, X’PertHighScore and the International Centre for Diffraction Data PDF-2 (ICDD) database). During the XRD measurement, the following parameters were used: 40 kV and 15 mA, step size 0.0435$^{\circ }$, 2$\theta$ range from 3 to 60, time per step 299.575 s. For crystalline phase identification following, ICDD entries were used-#026-1056 for OCP and #009-0348 for $\alpha$-TCP. The Fourier-transform infrared spectrometer Nicolet iS 50 (Thermo Scientific, Waltham, MA, USA) was used in transmission mode with the potassium bromide (KBr) pellet method. The FTIR spectra were recorded in the range of 4000–400 cm$^{-1}$, with 64 scans at a resolution of 4 cm$^{-1}$.

The XRD pattern served as a tool for identifying the three primary crystalline phases, their ratios varying with hydrolysis time: $\alpha$-TCP, DCPD, and OCP. The analysis pipeline will focus on the initial and final time points of the scaled-up synthesis (1 h, 24 h, 144 h, and 180 h), while additional specifics can be found in^9^. The XRD patterns gradually transition from the $\alpha$-TCP phase through DCPD to the OCP phase. Initially, only $\alpha$-TCP was observed, consistent with the ICDD pattern (#009-0348) of $\alpha$-TCP. The most prominent peaks were located at 12.1 and 30.7 $2\theta$ degrees, with double peaks around 22.8 $2\theta$ degrees and approximately 34 $2\theta$ degrees. After completion of the synthesis, the prominent peaks for OCP remained. While the overlap in the XRD pattern between 25–35 $2\theta$ degrees poses a challenge in distinguishing between HAp and OCP, peculiar reflections at lower angles confirmed the OCP phase. Specifically, the XRD pattern of OCP (seen in the final stages of the synthesis at 144 h and 180 h) exhibits a unique low angle (100) peak at $2\theta$ 4.72$^{\circ }$, along with a doublet at $2\theta$ 9.44$^{\circ }$ (200) and 9.77$^{\circ }$ (010). The key observations in the IR spectrum were the following: within the initial 24 h of synthesis, the most prominent bands of $\alpha$-TCP were prevalent. The most intense bands in the $\alpha$-TCP spectrum were identified in the ranges of 1300–900 cm$^{-1}$ and 700–500 cm$^{-1}$, associated with the vibrations of $\hbox {PO}_{4}^{3-}$. The concentrated peaks within these domains facilitated the easier distinction of $\alpha$-TCP from other calcium phosphates. As the synthesis progressed towards its final stages (at 144 h and 180 h), distinct features emerged. The $\nu$3 stretching mode of $\hbox {PO}_{4}^{3-}$ and $\hbox {HPO}_{4}^{2-}$, detected at 1077 cm$^{-1}$, 1093 cm$^{-1}$, and 1121 cm$^{-1}$, along with the subtle but characteristic line of OCP $\hbox {HPO}_4\,(6)\,[\hbox {P}-(\hbox {OH})]$ stretching at 917 cm$^{-1}$, and O–H in-plane bending at approximately 1295 cm$^{-1}$, became discernible. The $\hbox {PO}_{4}^{3-}$ $\nu$4 domain in the FTIR spectrum of OCP encompassed absorbance bands at 524, 560, 601, and 627 cm$^{-1}$.

### Computational resources

All numerical experiments of the proposed analysis pipeline were demonstrated on commodity hardware (a laptop computer equipped with Intel i5 CPU and 16 Gb RAM). This choice ensured the reproducibility of the current analysis sequence by other groups or researchers because it does not require cloud or cluster computing resources. Custom Python functions processed and analyzed the data upon importing the spectra as CSV files.

### XRD and FTIR signals pre-processing

**Figure 5:**
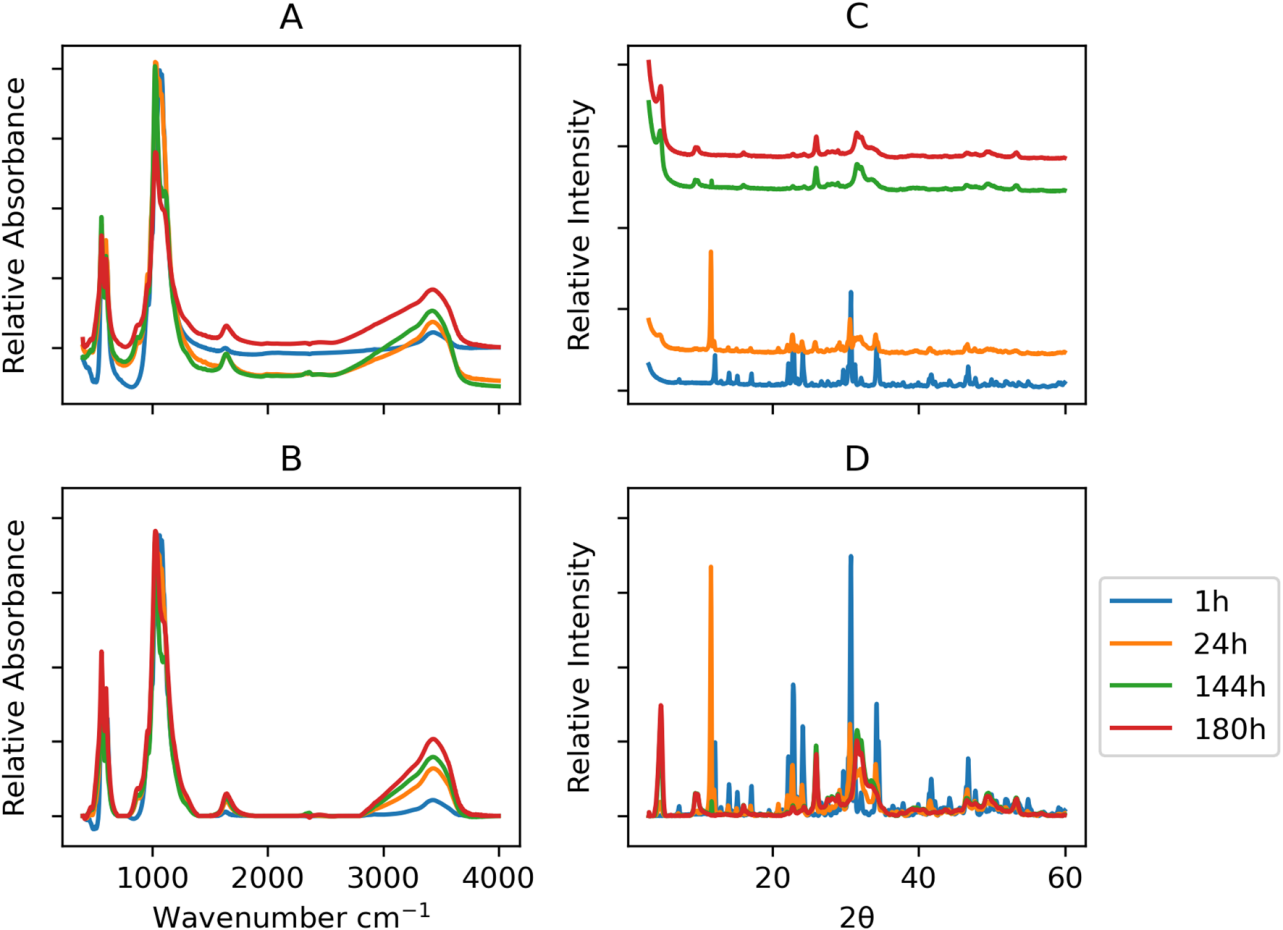
Effect of standardization and baseline correction on XRD and FTIR signals: (**A**) raw FTIR signals, (**B**) FTIR post-processed signals, (**C**) raw XRD signals, (**D**) XRD post-processed signals. The x-axes between vertical plots are shared.

Several techniques could be applied to spectroscopic spectra to adjust deviations from the ordinate axis^38^. In the current study, the FTIR baseline correction was performed by preselecting a few wavenumber ranges supposed to pertain to the baseline and to model the signals inside these regions with principal component analysis, then interpolating the first component’s loadings over the spectra and finally adapting the baseline of the spectra by the multiplication between principal component scores and first component’s interpolated loadings^39^. Afterward, signals were standardized, centering the point values on the median (rather than the mean) and the distances divided by the interquartile range (rather than standard deviation). This standardization methodology should keep the same degree of values between peaks and baseline values as found in the original input. Indeed, peaks could be interpreted as “outliers” compared to baseline points if standardized by the mean and standard deviation that might shrink the data range. As reported in other literature manuscripts, the data bounds were preserved using the median and interquartile range^40–42^. Furthermore, the data range was normalized between zero and one. For XRD, the signals were smoothed by a Hamming window of five points^43^ and baseline corrected with rubberband fitting^44^. Standardization and normalization were the same as applied to FTIR signals (Fig. 5). After processing, both XRD and FTIR signals resulted in a compatible range; thus, features extracted from the peaks could be aggregated and do not require further manipulation.

### XRD and FTIR peak identification and modeling

The primary XRD maxima and FTIR absorbance bands were corroborated according to the literature data: articles attributing FTIR wavenumbers relevant for OCP or $\alpha$-TCP determination were^13,14,45–47^, and for XRD phase composition, the maxima were selected according to angles suggested following the previous body of knowledge in the field^45,48–53^. All parameters from the literature were marked as vertical lines in the Figures SM1 and SM2, and peak identification on actual signals was adjusted according to a tolerance margin accounting for peak shifts^54,55^. The values found in the literature matched the signals, as detailed in^9^.

The specific vibrations of absorbance bands extracted from the literature are reported in Figure SM1. Valleys were searched in the points surrounding the peaks by inspecting the sign change in the first derivative (from negative to positive), and checking if the second-order derivative was negative. The noise level was estimated as the average of the baseline signal and used to exclude any peak-to-valley distance below the noise threshold. Each peak was modeled according to Gaussian, Lorentzian, or Voigt shapes; however, no Voigt shapes were observed. To design appropriate curves, the valleys of each fitted shape were lengthened from the edges using the tangent line upon reaching the zero absorption height.

Each XRD pattern was linearly detrended to remove any spurious deviation from the x axis. Figure SM2 displays the maxima detected from the current literature for possible initial and final phases. Then each peak was associated with the corresponding valley with the same procedure applied to FTIR, measuring the local minima preceding and coming after the peak.

The signals at 1 h and 24 h were labeled as the “Initial phase”, abbreviated as “I”, whereas signals at 144 h and 180 h were the latter synthesis phases, called the “Final stage”, and abbreviated as “F”. Initial phase wavenumbers identifying absorbance bands from FTIR spectra analysis were 563, 585, 597, 954, 984, 1025, and 1039 cm$^{-1}$; those for XRD were at 12.10, 22.21, 22.72, 24.10, 29.65, 30.6, 31.25 2$\theta$ angles. Final phase wavenumbers characterizing FTIR absorbance bands were 560, 601, 872, 962, 1023, 1077, 1108, and 1295 cm$^{-1}$; the angles for XRD were 4.72, 9.44, 9.76, 16.10, 26, 31.55, 32.59, 33.52. The angles and wavenumber values of each peak were illustrated in Figures SM3a and SM3b, in relation to class membership. Using these strip plots, one could note the shifts in XRD or FTIR associated with each class; despite a few peculiar patterns, most data points only fluctuate a few values along the x-axis.

#### XRD and FTIR peak modeling

Utilizing the peak height and distance from the valley, each peak was represented by a Gaussian, Lorentzian, or Voigt shape. The best matching curve fitting was selected to minimize the variance error. Tables SM1 and SM2 collect information about each peak fitted shape (“G” stands for Gaussian or “L” is for Lorentzian) .

From each fitted curve, nine features were computed:

- Normalized absorbance or intensity at the peak (also known as height of the peak) from zero
- Dispersion of the fitted distribution measured at 2 and 3 standard deviations
- Area under the fitted curve
- Kurtosis (descriptor of tailedness of the fitted shape)
- Skewness (descriptor of asymmetry of the fitted shape)
- Ratio between the normalized absorbance or intensity of a peak and a valley
- Ratio between peak height and width
- Amplitude at half width

All nine features from XRD and FTIR were merged in a unique dataset of 56 rows, shuffling the entries row-wise. At the same time, the two mentioned above class labels were associated with each instance: “I” meant the early stages of OCP production (present phase: $\alpha$-TCP at 1 h and 24 h), and “F”, the latter phases of the OCP transformation enclosing 144 h and 180 h time points. In this way, each row of the joint dataset was linked to a class summarizing the phase of the OCP synthesis (Table 2). Moreover, each row representing the features extracted from the fitted models of the XRD and FTIR peaks had a corresponding theta or wavenumber value.

**Table 2:** Class labels associated to each time point of OCP production.

Time	Class label	Class instances	XRD instances	FTIR instances
1 h and 24 h	Initial	26	14	12
144 h and 180 h	Final	30	14	16

### Feature selection

Not all nine features derived from the XRD and FTIR peak fitting might be meaningful in establishing peculiar characteristics of the time evolution during OCP production. For example, a few of them could be redundant or less informative than others. For this reason, a machine learning methodology has been employed to score each feature and retain only those most important in determining the time frame of OCP phase shifts. In other terms, features were scored in their ability to distinguish the class labels. With the recursive feature elimination procedure, a classifier is continuously trained on all features removing the one that contributes less than others to the classification results, as shown for nanomaterials toxicity prediction^56^. At the end of the procedure, each feature is ranked by the times it contributes to the best outcomes using a Random Forest classifier coded to account for class imbalances^57^. Additionally, cross-validation was chosen as the training method to provide a complete evaluation of all instances and enhance generalization (3-fold stratified cross-validation)^58^. The less essential dataset attributes for phase discrimination were the peak’s kurtosis and the height/width ratio.

### Spatial filtering

In current implementation the reduced dataset containing the seven most significant attributes was inputted to a custom Python function acting as spatial filter. The spatially filtered data matrix to discriminate the two classes could be written as

1 $$\begin{aligned} F=W^TE \end{aligned}$$

with *F* the surrogate, spatially filtered points, *E* the original signal feature array of *N* samples, and *W* the spatial filters. The matrix *W* contains the eigenvectors corresponding to the first eigenvalue and the last one: through general eigen-decomposition it could be possible to maximize the ratio of the projected covariance of one condition compared to the other, highlighting the discriminative patterns optimized on the variance of the classes. In the proposed approach, the covariance matrices for the two classes $C_{class1}$ and $C_{class2}$ could be computed by simultaneous diagonalization

2 $$\begin{aligned}{} & {} C_{class1} = \frac{E_{class1}\, E_{class1}^{T}}{N_{class1}} \end{aligned}$$

3 $$\begin{aligned}{} & {} C_{class2} = \frac{E_{class2}\, E_{class2}^{T}}{N_{class2}} \end{aligned}$$

4 $$\begin{aligned}{} & {} C = C_{class1} + C_{class2} = P_{0} + D + P_{0}^{T} \end{aligned}$$

with $P_{0}$ the matrix of eigenvectors, whereas *D* represents the diagonal matrix of eigenvalues of *C*. In *D* the eigenvalues are sorted in descending order to facilitate the identification of the first and the last one. Solving by applying the generalized eigenvalue problem, and considering that the eigenvectors are the same for both classes

5 $$\begin{aligned} C_{class2}^{-1}\, C_{class1} = P\, D\, P^{-1} \end{aligned}$$

which is equivalent to

6 $$\begin{aligned}{} & {} D = P\, C_{class1}\, P^{T} \end{aligned}$$

7 $$\begin{aligned}{} & {} I = P\, C_{class2}\, P^{T} \end{aligned}$$

with *I* the identity matrix such that $I = D_{class1} + D_{class2}$.

The first and last eigenvectors of *P* according to the eigenvalues could be selected as spatial filters. Similar method based on eigenvectors was applied on other disciplines to evaluate spatial dependence^26,28,59–61^.

### k-PCA

Kernel principal component analysis (k-PCA) is an extension of the classical principal component analysis technique^62^; in many real-world applications, data may not be linearly separable, and conventional PCA may not capture the underlying structure effectively. Kernel PCA addresses this limitation by implicitly mapping the input data into a high-dimensional feature space, where it becomes linearly separable. The key idea behind Kernel PCA is to use a kernel function to implicitly transform the input data into a higher-dimensional space, where linear techniques can be applied more effectively^63^. The “kernel trick” captures complex, nonlinear relationships in the data. The most commonly used kernel functions include the polynomial kernel, radial basis function kernel, and sigmoid kernel. The k-PCA procedure initially calculates the similarity or distance between each pair of data points based on the chosen kernel function. Afterward, the kernel matrix is transformed to ensure the data is centered in the feature space. It is performed by subtracting the mean of each column and each row of the kernel matrix from the corresponding elements; this operation centers the data around zero in the feature space. Then, the eigenvectors and eigenvalues of the centered kernel matrix are computed. These eigenvectors represent the directions in the high-dimensional space that capture the most variance in the data. Finally, the data is projected onto the principal components obtained from the eigenvectors. In kernel principal component analysis, the data’s measurement unit remains unchanged as the kernel trick is implicitly applied to map the data into a higher-dimensional space. In Figs. 1, 2, and 3, the two-component k-PCA was applied, with the axes representing the directions in the high-dimensional feature space that capture the most variance after the data has been implicitly mapped using a chosen kernel function. Unlike standard PCA, where the axes represent the principal components that are linear combinations of the original features, the axes in k-PCA represent nonlinear combinations of the original features.

### Supplementary Information

Supplementary Information.

## Data Availability

The datasets generated and/or analysed during the current study are available from the corresponding author on reasonable request.
