# Supplementary material for: Fusion and classification algorithm of octacalcium phosphate production based on XRD and FTIR data
Source: Sci Rep. 2024 Jan 17;14:1489. doi: 10.1038/s41598-024-51795-0 (PMC10794451; doi:10.1038/s41598-024-51795-0)
Supplement: Supplementary file 1 — Supplementary Information. [file 41598_2024_51795_MOESM1_ESM.pdf]

# Fusion and classification algorithm of octacalcium phosphate production based on XRD and FTIR data

**Mauro Nascimben[1,2,*], Ilijana Kovrlija[3], Janis Locs[3,4], Dagnija Loca[3,4], and Lia Rimondini[1]**

[1]Center for Translational Research on Autoimmune and Allergic Diseases - CAAD, Department of Health Sciences, Università del Piemonte Orientale UPO, Novara, 28100, Italy
[2]Enginsoft SpA, Padua, 35129, Italy
[3]Rudolfs Cimdins Riga Biomaterials Innovation and Development Centre, Institute of General Chemical Engineering, Faculty of Materials Science and Applied Chemistry, Riga Technical University, Riga, Pulka 3, LV-1007, Latvia
[4]Baltic Biomaterials Centre of Excellence, Headquarters at Riga Technical University, Riga, Latvia
[*]m.nascimben@enginsoft.com

## SUPPLEMENTARY MATERIALS

## XRD and FTIR peak identification and modeling

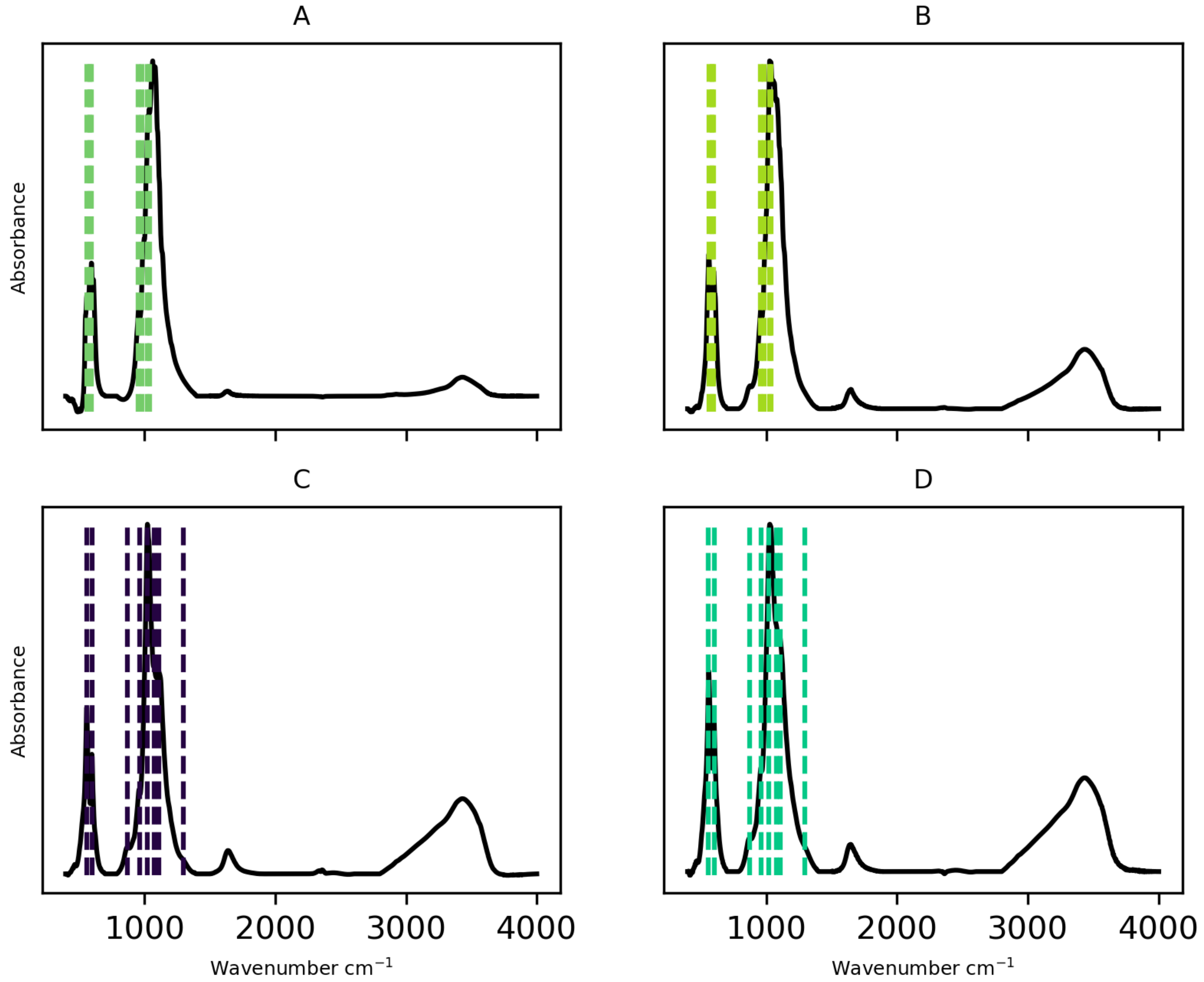


**Figure SM1.** Characteristic band positions based on the literature on each FTIR spectrum of initial (I) and final (F) stage of OCP synthesis: A) FTIR spectra of 1h time point – initial stage; B) FTIR spectra of 24h time point – initial stage; C) FTIR spectra of 144h time point – final stage; D) FTIR spectra of 180h time point – final stage.

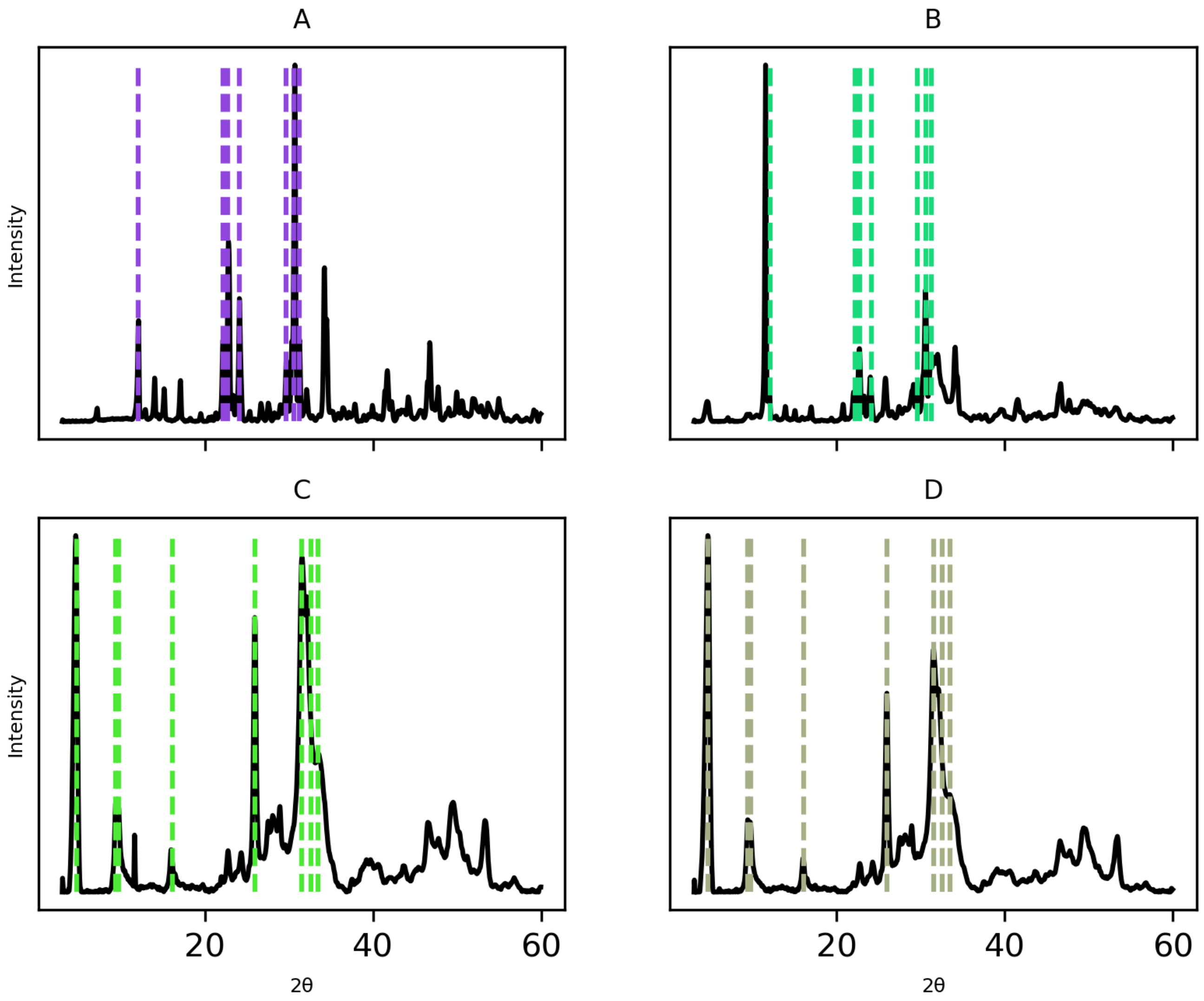


**Figure SM2.** Identified peaks were marked on each XRD signal of initial (I) and final (F) stage of OCP synthesis: A) XRD signal of 1h time point – initial stage; B) XRD signal of 24h time point – initial stage; C) XRD signal of 144h time point – final stage; D) XRD signal of 180h time point – final stage.

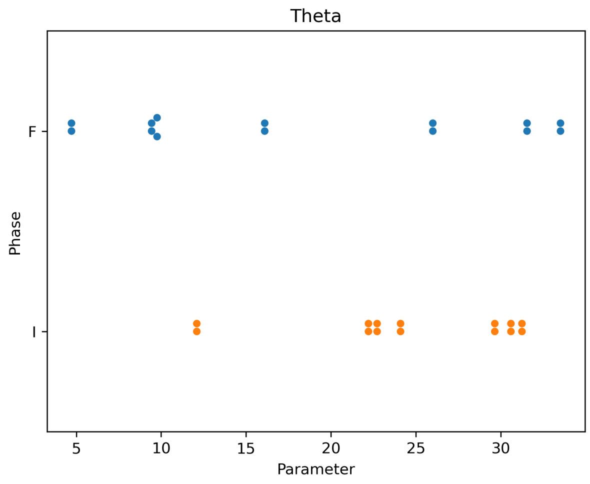


**(a)** Class membership of XRD $\theta$ angles.

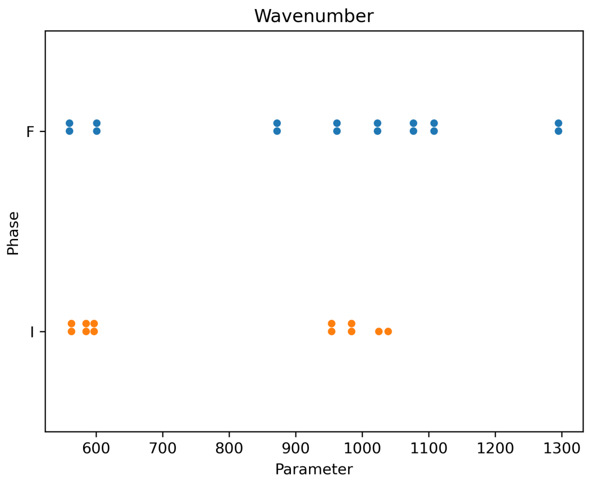


**(b)** Class membership of FTIR wavenumber values.

**Figure SM3.** Relation between production phases and XRD or FTIR values of each detected peak. Phase stands for initial or final, whereas Parameter means XRD angle or FTIR wavenumber.

## XRD and FTIR peak modeling

| Wavenumber | Phase | Fitting | Time |
|---|---|---|---|
| 560 | F | G | 180h |
| 560 | F | L | 144h |
| 563 | I | G | 24h |
| 563 | I | G | 1h |
| 585 | I | G | 1h |
| 585 | I | G | 24h |
| 597 | I | G | 1h |
| 597 | I | L | 24h |
| 601 | F | G | 144h |
| 601 | F | G | 180h |
| 872 | F | L | 180h |
| 872 | F | L | 144h |
| 954 | I | G | 1h |
| 954 | I | G | 24h |
| 962 | F | G | 144h |
| 962 | F | G | 180h |
| 984 | I | G | 1h |
| 984 | I | G | 24h |
| 1023 | F | G | 180h |
| 1023 | F | L | 144h |
| 1025 | I | G | 1h |
| 1039 | I | G | 1h |
| 1077 | F | G | 180h |
| 1077 | F | G | 144h |
| 1108 | F | G | 144h |
| 1108 | F | G | 180h |
| 1295 | F | G | 144h |
| 1295 | F | L | 180h |

**Table SM1.** FTIR curve fitting summary

| Angle | Phase | Fitting | Time |
|---|---|---|---|
| 4.72 | F | L | 144h |
| 4.72 | F | L | 180h |
| 9.44 | F | G | 180h |
| 9.44 | F | G | 144h |
| 9.76 | F | G | 144h |
| 9.76 | F | G | 180h |
| 12.10 | I | G | 24h |
| 12.10 | I | L | 1h |
| 16.10 | F | G | 144h |
| 16.10 | F | G | 180h |
| 22.21 | I | G | 24h |
| 22.21 | I | L | 1h |
| 22.72 | I | G | 1h |
| 22.72 | I | L | 24h |
| 24.10 | I | L | 1h |
| 24.10 | I | G | 24h |
| 26.00 | F | L | 144h |
| 26.00 | F | L | 180h |
| 29.65 | I | G | 24h |
| 29.65 | I | G | 1h |
| 30.60 | I | G | 24h |
| 30.60 | I | G | 1h |
| 31.25 | I | G | 1h |
| 31.25 | I | G | 24h |
| 31.55 | F | L | 144h |
| 31.55 | F | L | 180h |
| 33.52 | F | G | 144h |
| 33.52 | F | G | 180h |

**Table SM2.** XRD curve fitting summary
